# Comparative Study of Retinal Vessel Segmentation Based on Global Thresholding Techniques

**DOI:** 10.1155/2015/895267

**Published:** 2015-02-22

**Authors:** Temitope Mapayi, Serestina Viriri, Jules-Raymond Tapamo

**Affiliations:** ^1^School of Mathematics, Statistics & Computer Science, University of KwaZulu-Natal, Durban 4000, South Africa; ^2^School of Engineering, University of KwaZulu-Natal, Durban 4000, South Africa

## Abstract

Due to noise from uneven contrast and illumination during acquisition process of retinal fundus images, the use of efficient preprocessing techniques is highly desirable to produce good retinal vessel segmentation results. This paper develops and compares the performance of different vessel segmentation techniques based on global thresholding using phase congruency and contrast limited adaptive histogram equalization (CLAHE) for the preprocessing of the retinal images. The results obtained show that the combination of preprocessing technique, global thresholding, and postprocessing techniques must be carefully chosen to achieve a good segmentation performance.

## 1. Introduction

Diabetic retinopathy (DR) accounts for about five percent of the causes of blindness globally, representing almost five million blind as stated by World Health Organization [1]. An early detection of DR is ensured through the regular examination of retinal images in diabetic patients, thus reducing the incidence of blindness cases. Automatic vessel segmentation has a great potential to assist ophthalmologists in the early detection of DR [2].

There have been various works done on the segmentation of vessels in retinal images. These works can be classified into two major categories. The first category is the unsupervised methods. This comprises vessel tracking [3–5], matched filter responses [6–8], morphology-based techniques [9, 10], and locally adaptive thresholding [11]. The second category is the supervised methods. This category requires manually labeled images for training. This includes the use of neural networks [12], Bayesian classifier [13],* k*-nearest neighbor classifier [14], and SVM classifier [10, 15], for the classification of the image pixels as either blood vessel or background tissue pixels. The method proposed in this paper belongs to the unsupervised method.

Chaudhuri et al. [6] implemented a matched filter by initially approximating the intensity of gray level profiles of the cross sections of retinal vessels using a Gaussian shaped curve. An Otsu thresholding technique was further applied to the matched filter response image to segment the retinal vessels. Hoover et al. [8] segmented retinal vessels by applying a threshold probing technique combining local vessel attributes with region-based attributes on matched filter response (MFR) image. Compared to [6] where a basic thresholding of an MFR was used, the method proposed by [8] reduced the false positive rate by as much as 15 times. Zhang et al. [16], having identified that the general matched filter responds to both vessels edges and nonvessel edges, extended the general matched filter with the first-order derivative of the Gaussian properties of the retinal vessels. Martinez-Perez et al. [17] applied the combination of scale space analysis and region growing to segment the vasculature. The technique proposed in [17] was, however, unable to segment the thin vessels. Zana and Klein [18] implemented a vessel segmentation method based on the use of mathematical morphology. Although the result achieved in [18] was good, the vascular structures were not always connected to one another. Jiang and Mojon [11] implemented an adaptive local thresholding based on a verification-based multithreshold probing scheme. The proposed technique in [11] was, however, faced with the limitations of some unconnected vascular structures and the inability to detect the thinner vessels.

Moment features were used by Marín et al. [19] for vessel segmentation. A 7D vector composed of gray level and moment invariants-based features for pixel representation was computed, while a neural network classifier is used for the vessel segmentation. Soares et al. [13] generated a feature vector computed from the measurements at different scales of two-dimensional (2D) Gabor wavelet transform on each pixel. Bayesian classifier with Gaussian mixtures was further used to classify the resulting feature space as either a vessel or nonvessel pixel. Staal et al. [14] implemented a ridge-based vessel segmentation method. The retinal image ridges which cooccur approximately with vessel centre-lines were extracted. Primitives in the form of line elements were further composed of the ridges. The feature vectors computed for every pixel were classified using a* k*-nearest neighbour classifier and sequential forward feature selection. Niemeijer et al. [20] implemented vessel segmentation method based on pixel classification. Each pixel of the green plane of the retinal image was used to construct a feature vector. Consequently, these feature vectors were trained using a* k*NN-classifier. A filtered output and the pixel values within a neighborhood were compared. The best results were obtained from the filter output. Niemeijer et al. [20] further did the comparative study of the proposed vessel segmentation technique with the techniques proposed in [11, 14]. Fraz et al. [21, 22] implemented a supervised segmentation technique based on ensemble classifier of bootstrapped decision trees for the segmentation retinal vessel network. Lupaşcu et al. [23] implemented a supervised segmentation technique for detecting vessels using Ada-Boost classifier. A feature vector comprising local and spatial properties of the vessels were generated from the responses of various filters (matched filters, Gabor wavelet transform, and Gaussian filter and its derivatives). Ada-Boost classier was further trained and used to classify each pixel as either vessel or nonvessel. Ricci and Perfetti [24] proposed two different automated vessel segmentations based on line operators. The best of the two segmentation methods constructed feature vector for supervised classification using a support vector machine.

Szpak and Tapamo [25] used gradient based approach and level set technique. The proposed technique in [25] was, however, unable to detect the thinner vessels. Vlachos and Dermatas [26] implemented a multiscale line-tracking combined with a morphological postprocessing technique. Wang et al. [27] proposed multiwavelet kernels and multiscale hierarchical decomposition for the segmentation of retinal vessels. Mendonça and Campilho [28] combined differential filters for center-line extraction with morphological operators for the detection of retinal vessel network. Xiao et al. [29] proposed a Bayesian method with spatial constraint with level set for the segmentation of retinal vessels. Yin et al. [30] implemented a probabilistic tracking-based method for vessel segmentation.

Lupascu and Tegolo [31, 32] trained a self-organizing map (SOM) on retinal images. The map was further divided into two classes using *k*-means clustering [31] and modified fuzzy *c*-means [32] techniques. The entire image is fed into SOM again and the class of the best matching unit on SOM is assigned to each pixel. A postprocessed technique based on hill climbing strategy on connected components was used to detect the vessel network. Saffarzadeh et al. [33] implemented a preprocessing phase based on *k*-means followed by the use of multiscale line operators for the detection of retinal vessel network. With the help of *k*-means, the visibility of the vessels was enhanced and the impact of bright lesions was reduced. The retinal vessels were finally detected using the line detection operator in three scales.

Setiawan et al. [34] used contrast limited adaptive histogram equalization (CLAHE) to enhance the green channel color retinal image in order to enhance color retinal fundus image. The enhancement was achieved using histogram manipulation to get the uniform distribution of the intensity of the green channel. Contrast limited adaptive histogram equalization spreads the intensity distribution and adjusts the intensity of the original image. The red, green, and blue channels were finally combined as an enhanced color retinal image. Phase congruency on the other hand is a technique that is not affected by uneven illumination and contrast of the retinal image. A bank of log-Gabor filters was used by Kovesi to compute the phase congruency of an image and a binary segmentation was obtained by universal thresholding [35, 36].

Amin and Hong [37] implemented the detection of retinal blood vessels using phase congruency at an high speed. Although the technique performed well in terms of speed, there is a need for a higher accuracy rate and a dynamically computed thresholding approach. Tagore et al. [38] used phase congruency to improve the contrast of vessel segments against the retinal background. A hierarchical clustering based histogram thresholding was then used to segment the contrast enhanced vessels. In related development, vessels cross-sectional profiles in the Fourier domain were represented and characterize using phase congruence by Zhu [39]. A bank of Gabor filter was used to transform the input image. The performance of the proposed technique in [39] was only described using visual results.

Although global thresholding technique has been used in [6], it has, however, been said to be inefficient for the retinal vascular segmentation [8, 11]. This might have also resulted from certain limitations of the preprocessing phase [16]. In order to effectively produce good vessel segmentation, there is a need for an efficient preprocessing phase to enhance the vessels, good global thresholding technique, and an efficient postprocessing technique. This paper presents a study on the use different global thresholding techniques combined with different preprocessing and postprocessing techniques. The rest of this paper is organized as follows.  Section 2 describes the methods and techniques used in this study.  Section 3 explains the experimental setup, results, and discussion, while the conclusion is summarized in Section 4.

## 2. Methods and Techniques

Retinal fundus images are often characterized by noise due to illumination and contrast variation. Due to this, the use of global thresholding techniques for the detection of vessels in these noisy retinal images becomes challenging. In order to solve this problem, the need for an efficient preprocessing technique is highly desirable. This section describes the two different preprocessing techniques and the different filtering techniques which are used to enhance the vessels. The different thresholding techniques and postprocessing techniques used in this paper are also described in this section. For the purpose of simplification, we group these techniques into two major approaches, namely, CLAHE global-based thresholding approach and phase congruence global-based thresholding approach as described in Algorithms 1 and 3.

(*1) Preprocessing Phase*. The different techniques used in the preprocessing phase are described below.

**Figure 1:**
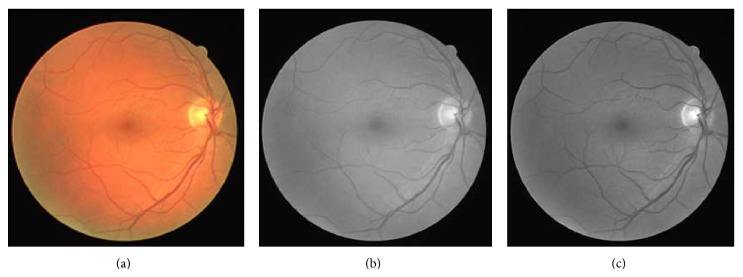
(a) Colored retinal image (b) Gray Scale Retinal Image. (c) Green channel of the colored retinal image.

(a) CLAHE: CLAHE algorithm is used for partitioning the image into contextual regions and it applies the histogram equalization to each one.  Figure 1 shows the colored, the gray scale, and the green channel of the retinal fundus image. CLAHE computes the local histogram at each pixel of the retinal image and performs histogram clipping, histogram renormalization, and output pixel mapping to an intensity proportional to its rank within the histogram. Given that *h*_*i*_ is the histogram bin and (*m* × *m*) is the contextual region, the rank *r*_*p*_ for a pixel with intensity *p* is computed as follows:

(1) rp=∑k=0Nmax⁡(0,hk−β)m×m‍∑i=0pmin⁡(β,hi)+p+1hhhh×∑k=0Nmax⁡(0,hk−β)m×m‍ ×m×m−1,

where the clip limit *β* determines the contrast enhancement limit and ∑_*i*=0_^*p*^min⁡(*β*, *h*_*i*_) describes the rank in a clipped histogram. Since each region will have a different number of clipped pixels, it is, however, beneficial to redistribute the part of the histogram that exceeds the clip limit *β* evenly among all histogram bins to normalize the ranks computed in different regions. This normalization is provided by ∑_*j*=0_^*p*^((∑_*k*=0_^*N*^max⁡⁡(0, *h*_*k*_ − *β*))/(*m* × *m*)), where *h*_*k*_ is the histogram bin in the different region. The rank of intensity *i*_in_ at (*x*, *y*) is computed and scaled to produce a fractional rank *r*, such that 0.0 ≤ *r* ≤ 1.0.

The output intensity level *i*_out_ is then computed in some grey scale ranging between *i*_1_ and *i*_2_ as follows:

(2) $$\begin{array}{c} i_{\text{out}}=i_{1}+r\times \left(i_{2}-i_{1}\right). \end{array}$$

(b) The phase congruence model proposed in [35, 36] has been very promising in the detection of object boundary in the presence of noise. The green channel is enhanced using phase congruence to minimize retinal image noise due to nonuniform illumination and contrast. Phase congruency is computed as follows:

(3) $$\begin{array}{c} \text{PC}\left(x\right)=\frac{\left|E\left(x\right)\right|}{\sum_{t}A_{t}\left(x\right)},\;0\leq \text{PC}\left(x\right)\leq 1, \end{array}$$

where *A*_*t*_(*x*) is the amplitude, *ϕ*_*t*_(*x*) is the phase, and |*E*(*x*)| is the local energy, given that

(4) $$\begin{array}{c} E\left(x\right)=\underset{t}{\sum }A_{t}\left(x\right)\cos\left(\phi_{t}\left(x\right)-\bar{\phi }\left(x\right)\right). \end{array}$$

**Figure 2:**
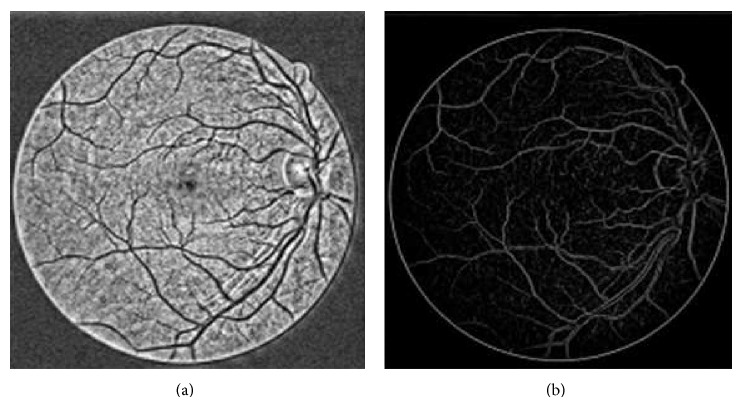
(a) Preprocessed retinal image using CLAHE (b) Preprocessed retinal image using phase congruence.

In order to apply phase congruence to images, (3) is modified to be as follows:

(5) $$\begin{array}{c} \text{PC}\left(x,y\right)=\frac{\sum_{\theta }\sum_{t}W_{\theta }\left(x,y\right)\left\lfloor A_{t,\theta }\Delta \phi_{t,\theta }\left(x,y\right)-T_{\theta }\right\rfloor }{\sum_{\theta }\sum_{t}A_{t,\theta }\left(x,y\right)+ɛ}, \end{array}$$

where (*x*, *y*) is the position of the pixel in the green channel of the retinal image, while *t* and *θ* are the given scale and orientation, respectively. *W*_*θ*_ is the weighing factor for the distributed frequency, while *T*_*θ*_ estimates the image noise. The energy is computed using *A*_*t*,*θ*_Δ*ϕ*_*t*,*θ*_(*x*, *y*), while *ɛ* is added to the denominator such that the divisor will be nonzero. The visual results of both CLAHE and phase congruence preprocessing techniques can be seen in Figure 2.

(c) Filters: the resulting images from CLAHE preprocessing technique are still affected to some extent by noise. In order to further enhance the retinal images, different filters are considered. The different filters considered are adaptive filter, average filter, and Gaussian filter. The combination of average filter and Gaussian filter was also used to further enhance the output of CLAHE preprocessing technique. Each of these different filtering approaches was considered in order to investigate their suitability for further enhancement of the retinal image. In related development, the resulting images from phase congruence were also enhance using average filter. The performance of each of the filtering approaches was, however, measured after the final vessel segmentation. The visual results from DRIVE database can be seen in Figures 4, 5, and 7 and those of STARE database in Figures 11 and 12.

(*2) Global Thresholding*. Automatic thresholding is potentially useful to dynamically select an optimal gray level threshold value for the segmentation of retinal vessels in the image from the background tissue based on their intensity distribution. The different global thresholding techniques studied in this paper are as follows.

**Figure 3:**
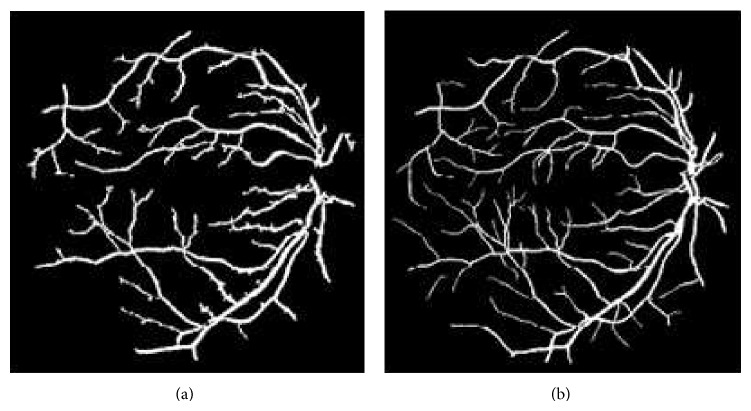
(a) Segmented retinal vessels using CLAHE preprocessing with Otsu threshold. (b) DRIVE database gold standard.

(a) Otsu thresholding: global thresholding technique based on Otsu [40] is used on the results computed from phase congruence and CLAHE with filters for the initial estimation of the vessel network. The threshold that minimizes the intraclass variance as a weighted sum of variances of the two classes is explored in Otsu's method. The weighted sum of variances of two classes is expressed as follows:

(6) $$\begin{array}{c} \sigma_{\omega }^{2}\left(t\right)=\omega_{1}\left(t\right)\sigma_{1}^{2}\left(t\right)+\omega_{2}\left(t\right)\sigma_{2}^{2}\left(t\right), \end{array}$$

such that weights *ω*_*i*_ describe the probabilities of the two classes separated by a threshold *t* and *σ*_*i*_^2^ variances of the classes. The class probability *ω*_1_(*t*) is then computed from the histogram as follows:

(7) $$\begin{array}{c} \omega_{1}\left(t\right)=\overset{t}{\underset{0}{\sum }}p\left(i\right), \end{array}$$

while the class mean *μ*_1_(*t*) is computed as follows:

(8) $$\begin{array}{c} \mu_{1}\left(t\right)=\frac{\left[\sum_{0}^{t}p\left(i\right)x\left(i\right)\right]}{\omega_{1}}, \end{array}$$

such that *x*(*i*) is the value at the center of the *i*th histogram bin. *ω*_2_(*t*) and *μ*(*t*) can likewise be computed on the histogram for bins greater than *t*. Otsu further showed that minimizing the intraclass variance is the same as maximizing interclass variance; thus the desired threshold *σ*_*b*_^2^(*t*) is given as follows:

(9) $$\begin{array}{c} \sigma_{b}^{2}\left(t\right)=\sigma^{2}\left(t\right)-\sigma_{\omega }^{2}\left(t\right)=\omega_{1}\left(t\right)\omega_{2}\left(t\right){\left[\mu_{1}\left(t\right)-\mu_{2}\left(t\right)\right]}^{2}, \end{array}$$

where *μ*_1_(*t*) and *μ*_2_(*t*) are the means of the first and second group, respectively. The visual result of Otsu threshold on the image obtained from CLAHE preprocessing technique can be seen in Figure 3.

(b) ISODATA threshold selection: ISODATA threshold technique divides the histogram of the image output from phase congruence method into two using an initial threshold value *t*_0_. The threshold is computed as follows:

(10) $$\begin{array}{c} t_{h}=\frac{m_{1}+m_{2}}{2}, \end{array}$$

where *m*_1_ and *m*_2_ are the mean values of the two different parts of the histogram. This process continues until *t*_*h*_ ≈ *t*_*h*−1_.

(c) Inverse difference moment (IDM)-based binary thresholding: image signal statistics, particularly first- and second-order statistics, are good texture feature descriptors used for supervised segmentation techniques. Moments, first-order statistics, are concerned with individual image pixel properties while second-order statistics such as gray level cooccurrence matrix (GLCM) are concerned with individual pixel properties as well as the spatial interdependency of the two pixels at particular relative positions. The IDM texture information is computed using the GLCM of the gray scale of the retinal fundus image. The GLCM for the retinal fundus image is computed in the relative distance* “d”* between the pixel pair and their relative orientation “Φ” across four directions (horizontal: 0°, diagonal: 45°, vertical: 90°, and antidiagonal: 135°) as

(11) $$\begin{array}{c} C_{i,j}=\overset{M-1}{\underset{x=0}{\sum }}\;\overset{N-1}{\underset{y=0}{\sum }}\left(P\left\{V\left(x,y\right)=i,\;\;V\left(x\pm d\Phi_{1},y\pm d\Phi_{2}\right)=j\right\}\right), \end{array}$$

where *V*(*x*, *y*) = *i* means *i* is the gray level of the pixel (*x*, *y*), and *P* is defined as

(12) P(x)=1if  x  is  true0Otherwise.

The IDM feature across the different distances* “d”* and varying relative orientation* “*Φ*”* is defined as follows:

(13) $$\begin{array}{c} \text{ID}{\text{M}}_{\left(d,\Phi \right)}=\underset{i,j}{\sum }\frac{p_{\left(i,j\right)}}{\left(1+{\left(i+j\right)}^{2}\right)}, \end{array}$$

where *p*_(*i*,*j*)_ is the (*i*, *j*)th entry in a normalized gray scale spatial dependence matrix *C*_(*i*,*j*)_/*R*.

A multiscale IDM feature measurement across the varying distance* “d”* and relative orientation* “*Φ*”* is used in the computation of an IDM feature matrix as follows:

(14) $$\begin{array}{c} F=\left(\begin{array}{cccc} f_{11} & f_{12} & f_{13} & f_{14}\\ f_{21} & f_{22} & f_{23} & f_{24}\\ f_{31} & f_{32} & f_{33} & f_{34}\\ f_{41} & f_{42} & f_{43} & f_{44} \end{array}\right), \end{array}$$

where *f*_*ij*_ = IDM_*d*_*i*_,Φ_*j*__ with orientations (Φ_*j*_)_*i*=1,…,4_, such that Φ_1_ = 0°, Φ_2_ = 45°, Φ_3_ = 90°, and Φ_4_ = 135°, with distances (*d*_*i*_)_*i*=1,…,4_. The range measure of *F* is given below as

(15) $$\begin{array}{c} R_{\Phi }=\underset{1\leq j\leq 4}{\max}\left(f_{ij}\right)-\underset{1\leq j\leq 4}{\min}\left(f_{ij}\right), \end{array}$$

where 1 ≤ *i* ≤ 4 and *R*_Φ_ is a row vector containing the range of each column of matrix (*F*).

The threshold value that will be used for the binarization of the output image from the phase congruence and average filter is computed as follows:

(16) $$\begin{array}{c} T_{h}=\max\left(R_{\Phi }\right)+\text{mean}\left(R_{\Phi }\right). \end{array}$$

(*3) Postprocessing Phase*. The different techniques used in the postprocessing phase are described below.

(a) Median filtering and morphological opening: median filter is used to restore the connectivity of several vessel lines by revealing some hidden pixels that belong to vessel lines. It is also used to get rid of the remaining noisy pixels. The choice of applying a 2 × 2 median filter has a good performance. This is referred to as (MO) in Tables 4 and 5. This is followed by the use of morphological opening in removing part of the remaining noisy pixels. The use of morphological opening (MO) alone and the combination of morphological opening and median filter was used. This is referred to as (MOMF) in Tables 4 and 5.

(b) Morphological directional filtering and reconstruction: the morphological directional filtering described in [12] is used to handle the several misclassifications that still remained. Morphological openings with line structuring elements orientation in five various directions, namely, 0, 30, 60, 120, and 150 degrees, are used. This paper adopts length of 1 pixel to keep vessel like structures with length of greater or less than 1. A logical OR for the responses of the five different directions and morphological reconstruction were performed on the image to remove a few erroneous regions before producing the final vessels network. The (MOMF) described in (a) above is combined with morphological directional filtering and morphological reconstruction for the purpose of performance investigation. This is referred to as (ATC) in Tables 4 and 5.

## 3. Experimental Results and Discussions

Experiment was carried out using Matlab 2010a on an Intel Core i5 2410 M CPU, 2.30 GHz, with 4 GB of RAM. The proposed method was evaluated using the retinal images on the publicly available DRIVE [41] and STARE databases [8]. DRIVE database is made up of 40 images captured with the use of Canon CR5 camera with 24-bit gray scale resolution and a spatial resolution of 565 × 584 pixels. The 40 images were divided into two groups. The first group of the DRIVE images is a training set made up of twenty images. The second group is a testing set made up of twenty images. DRIVE database also provides gold standard images as the ground truth for vessel segmentation for the comparative performance evaluation of different vessel segmentation algorithms. STARE database on the other hand consists of retinal images captured with the use of TopCon TRV-50 fundus camera with 24-bit gray scale resolution and spatial resolution of 700 × 605 pixels. The database provides 20 coloured retinal images and 20 hand-labeled images as the ground truth for the comparative performance evaluation of different vessel segmentation algorithms.

The outcome of retinal vessel segmentation is a pixel-based classification result. Each pixel is either classified as vessel or background. Different events such as true positive (TP), true negative (TN), false positive (FP), and false negative (FN) take place during the pixel classification. An event is said to be TP if a pixel is correctly segmented as a vessel and TN when a pixel is correctly segmented as background. In related development, an event is said to be FN if a vessel pixel is segmented to be a background and a FP when a background pixel is segmented as a pixel in the vessel. The statistical performance measures commonly used for the evaluation of segmentation techniques are sensitivity, specificity, and accuracy. Sensitivity measure indicates the ability of a segmentation technique to detect the vessel pixels while specificity measure indicates the ability of a segmentation technique to detect background pixels. The accuracy measure, however, indicates the degree of conformity of the segmented retinal image to the ground truth. The measures are described in the equation below as

(17) $$\begin{array}{c} \text{Sensitivity}=\frac{\text{TP}}{\left(\text{TP}+\text{FN}\right)},\\ \text{Specificity}=\frac{\text{TN}}{\left(\text{TN}+\text{FP}\right)},\\ \text{Accuracy}=\frac{\left(\text{TP}+\text{TN}\right)}{\left(\text{TP}+\text{TN}+\text{FP}+\text{FN}\right)}, \end{array}$$

where TP is true positive, TN is true negative, FP is false positive, and FN is false negative.

Table 1 gives an overview of the parameter description and optimum parameter values of the phase congruence technique. In related development, different optimal values were empirically selected for the parameters used in CLAHE global thresholding approaches as described in Table 2.

**Figure 4:**
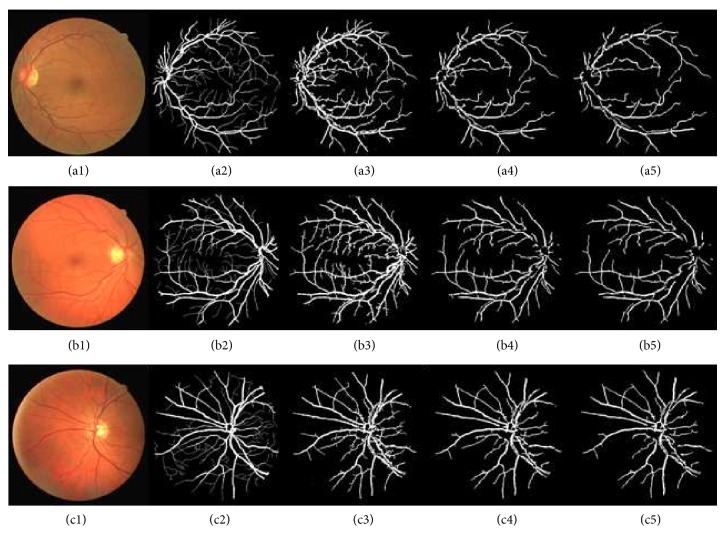
Shows retinal images and their segmentation results obtained through phase congruence using different global-based thresholding techniques. Images (a1), (b1), and (c1) are DRIVE database colored retinal Images. Images (a2), (b2), and (c2) are DRIVE database gold standards. Images (a3), (b3), and (c3) are images segmented using IDM-based threshold values while images (a4), (b4), and (c4) are images segmented using ISODATA threshold values. Images (a5), (b5), and (c5) are images segmented using Otsu threshold values.

Figure 4 shows retinal images and their segmentation results obtained through phase congruence using different global-based thresholding techniques on DRIVE database.  Figure 6 also shows the segmentation result obtained from a diseased retinal from DRIVE database using phase congruence combined with IDM thresholding technique.  Figure 12 shows the result of phase congruence-based global thresholding approach on STARE database.

**Figure 5:**
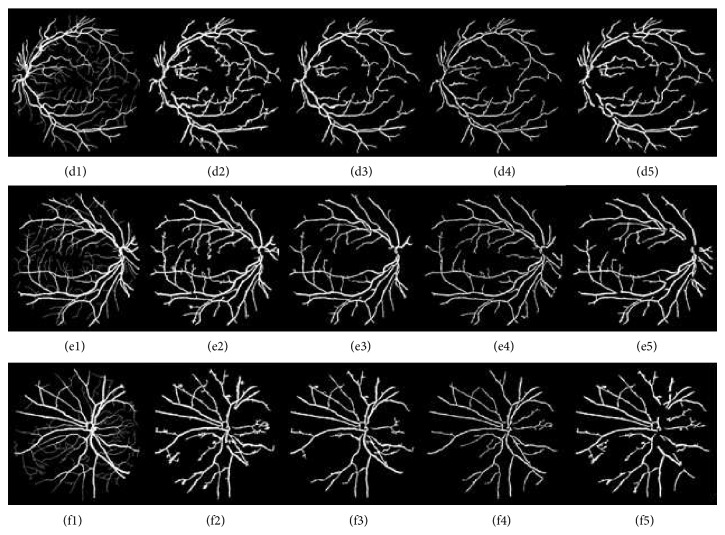
Shows different segmentation results obtained through CLAHE with different filters using Otsu thresholding technique. Images (d1), (e1), and (f1) are DRIVE database gold standards. Images (d2), (e2), and (f2) are images segmented using Otsu threshold with Gaussian filter. Images (d3), (e3), and (f3) are images segmented using Otsu threshold with average filter. Images (d4), (e4), and (f4) are images segmented using Otsu threshold with adaptive filter. Images (d5), (e5), and (f5) are images segmented using Otsu threshold with combination of average and Gaussian filters.

**Figure 6:**
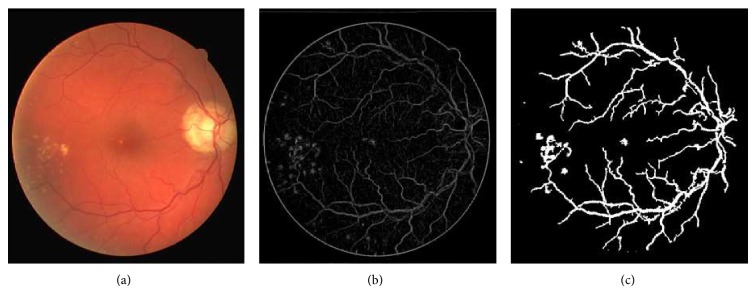
(a) Colored retinal image. (b) Preprocessed image using phase congruence. (c) Segmented retinal image containing vessel network and lesions.

**Figure 7:**
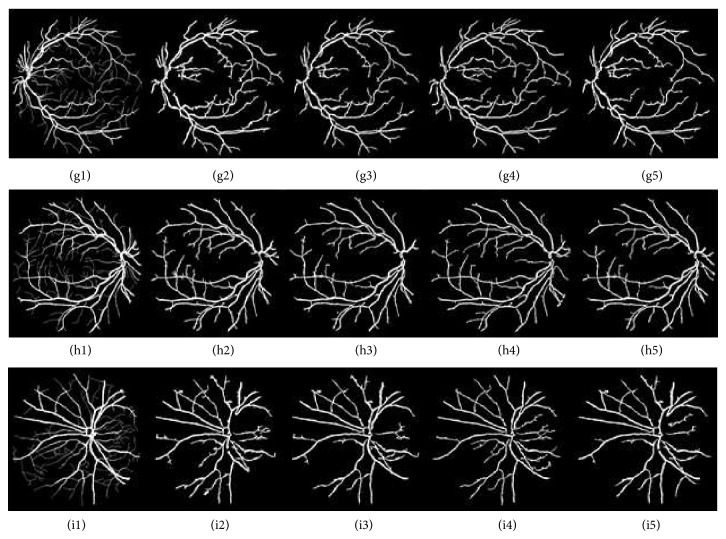
CLAHE combined with ISODATA thresholding technique. It shows the different segmentation results obtained through CLAHE with different filters using ISODATA thresholding technique. Images (g1), (h1), and (i1) are DRIVE database gold standards. Images (g2), (h2), and (i2) are images segmented using ISODATA threshold with Gaussian filter. Images (g3), (h3), and (i3) are images segmented using ISODATA threshold with average filter. Images (g4), (h4), and (i4) are images segmented using ISODATA threshold with adaptive filter. Images (g5), (h5), and (i5) are images segmented using ISODATA threshold with combination of average and Gaussian filters.

Figure 5 shows different segmentation results obtained through CLAHE combined with different filters using Otsu thresholding technique while Figure 7 shows different segmentation results obtained through CLAHE combined with different filters using ISODATA thresholding technique on DRIVE database.  Figure 11 also shows the result of CLAHE-based global thresholding approaches on STARE Database.

**Figure 8:**
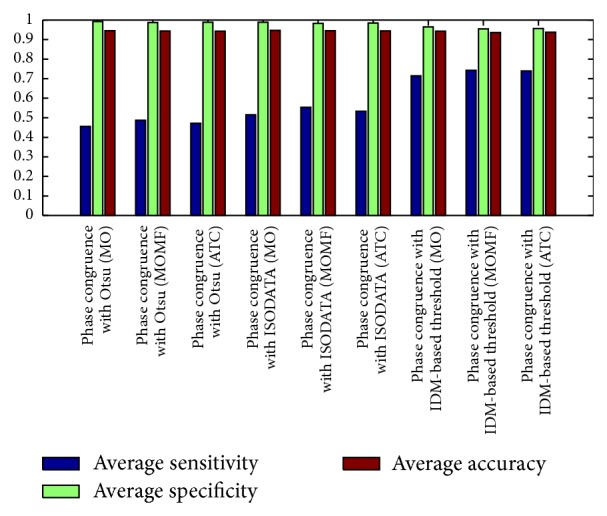
Measures the different phase congruence-based global thresholding approaches. It describes the average sensitivity, specificity, and accuracy of the segmentation results obtained through phase congruence using different global-based thresholding techniques. Phase congruence with IDM-based threshold, combined with (MO), gives the average accuracy of 0.94302, average sensitivity of 0.71520, and average specificity of 0.96496.

**Figure 9:**
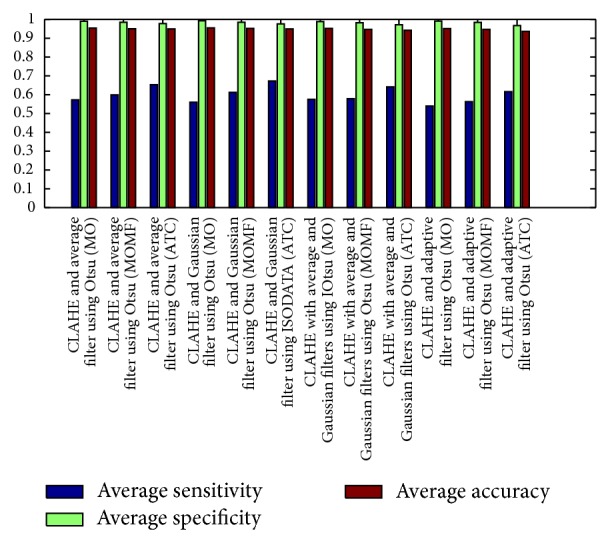
Measures of CLAHE combined with different filters using Otsu threshold. It describes the average sensitivity, specificity, and accuracy of the segmentation results obtained through CLAHE with Otsu thresholding using different filters. CLAHE with guassian filters, combined with (ATC), gives the best performance of an average accuracy of 0.94980, average sensitivity of 0.67290, and average specificity of 0.97651.

**Figure 10:**
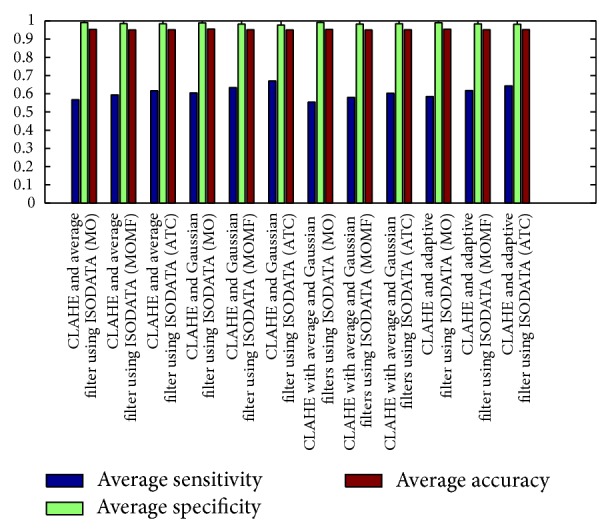
Measures of CLAHE combined with different filters using ISODATA threshold. It describes the average sensitivity, specificity, and accuracy of the segmentation results obtained through CLAHE with ISODATA thresholding using different filters. CLAHE with guassian filters, combined with (ATC), gives the best performance of an average accuracy of 0.94997, average sensitivity of 0.67011, and average specificity of 0.97695.

**Figure 11:**
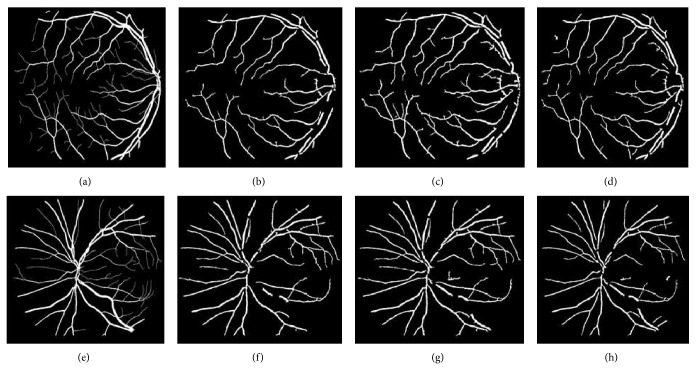
(a) and (e) are STARE database ground truth. (b) and (e) are images segmented using global threshold with adaptive filter. (c) and (f) are images segmented using global threshold with average filter. (d) and (g) are images segmented using global threshold with Gaussian filter.

**Figure 12:**
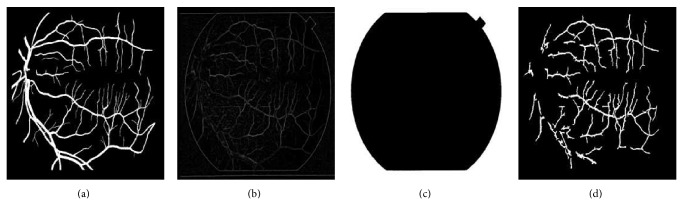
(a) STARE database ground truth. (b) Preprocessed image using phase congruence. (c) Retinal image mask. (d) Segmented vessel network using phase congruence-based global thresholding approach.

Figure 8 describes the average sensitivities, specificities, and accuracies of the segmentation results obtained from phase congruence-based global thresholding approaches while Figures 9 and 10 show the average sensitivities, specificities, and accuracies of the segmentation results obtained from CLAHE-based global thresholding approaches on DRIVE database.

Table 3 shows the performance of the different global thresholding techniques on DRIVE database. Although CLAHE-based global thresholding approaches have very good accuracies due to the accurate segmented vessels, they, however, possess lower sensitivities due to the inability to segment the thin vessels. CLAHE-based global thresholding approaches are at their best performance when all the postprocessing techniques are combined. The best average sensitivity and accuracy results of CLAHE with Gaussian filter using Otsu threshold are 0.67290 and 0.9498. The next in rank of CLAHE-based preprocessing combined with Otsu threshold is CLAHE with average filter giving the average sensitivity and accuracy results of 0.65349 and 0.9494. CLAHE with average and Gaussian filters gives the average sensitivity and accuracy results of 0.64159 and 0.94269 while CLAHE with adaptive filter gives the average sensitivity and accuracy results of 0.61596 and 0.93678. The best average sensitivity and accuracy results of CLAHE with Gaussian filter using ISODATA threshold are 0.67011 and 0.94997. CLAHE-based preprocessing combined with average filter gives the average sensitivity and accuracy results of 0.61630 and 0.95162 for the ISODATA threshold technique. CLAHE-based preprocessing combined with average and Gaussian filters gives the average sensitivity and accuracy results of 0.60265 and 0.95104 for the ISODATA threshold technique. CLAHE with adaptive filter also gives the average sensitivity and accuracy results of 0.64348 and 0.95209 for the ISODATA threshold technique.

The best results achieved using phase congruence-based global thresholding approaches are obtained using IDM-based thresholding compared to ISODATA and Otsu thresholding. Phase congruence combined with IDM-based thresholding has very good accuracies due to the accurate segmented vessels and possesses good sensitivities due to the ability to segment some thin vessels. It is, however, still unable to segment the thinnest vessels. The best average accuracy of 0.94302 and average sensitivity of 0.71520 are achieved using morphological opening postprocessing technique. The next in rank of phase congruence-based global thresholding approaches is IDM-based thresholding combined with all postprocessing techniques combined giving average accuracy and sensitivity results of 0.93772 and 0.73910. IDM-based thresholding combined with morphological opening combined with median filter gives average accuracy and sensitivity results of 0.93596 and 0.74247.

Phase congruence combined with IDM-based thresholding generally performed better than all the CLAHE-based global thresholding approaches. Phase congruence combined with IDM-based thresholding also gives better performance compared to Otsu and ISODATA thresholding combined with phase congruence. The performances of Otsu and ISODATA thresholding coupled with phase congruence are, however, at their best when morphological opening and median filter are combine for the post processing phase.

Tables 4 and 5 describe the performances of the best techniques from the different segmentation methods investigated in this paper and other previously published works using DRIVE and STARE databases.

Phase congruence combined with IDM-based thresholding using morphological opening postprocessing technique presents a higher average accuracy rates on DRIVE and STARE databases compared to the previously proposed phase congruence based technique by Amin and Hong [37]. Tagore et al. [38] achieves a lower average accuracy rate on DRIVE database but a higher average accuracy rate compared to the best phase congruence-based global thresholding approach presented in this paper. The technique proposed in [38], however, did not present the sensitivity and specificity measures. In related development, the phase congruence technique proposed by Zhu [39] discussed only the visual performance. Tables 4 and 5 also compare the results obtained in this paper with other results achieved in other literatures.

## 4. Conclusion

The performance of different vessel segmentation approaches based on combination of different preprocessing techniques, global thresholding, and postprocessing techniques has been investigated. It has also been shown that the combination of preprocessing technique, global thresholding, and postprocessing techniques must be carefully chosen to achieve a good segmentation performance. It is, however, important to state that the paper shows that sensitivity, specificity, and accuracy measures must all be high to ascertain a good segmentation performance. It was also shown that phase congruence combined with IDM-based thresholding generally performs better compared to phase congruence combined with ISODATA and Otsu threshold. Phase congruence combined with IDM-based thresholding is at its best on DRIVE database but did not have a better performance compared to the best of the CLAHE-based global thresholding approaches on STARE database. CLAHE-based global thresholding approaches were, however, shown to have maintained high accuracy rates across DRIVE and STARE databases. Although good accuracy and specificity rates were achieved, the sensitivity rate shows that global thresholding approach is still limited at efficiently segmenting the thin vessels. Our future work shall investigate the use of more robust segmentation techniques for the detection of both large and thin vessels in retinal images.

## Figures and Tables

**Algorithm 1:**
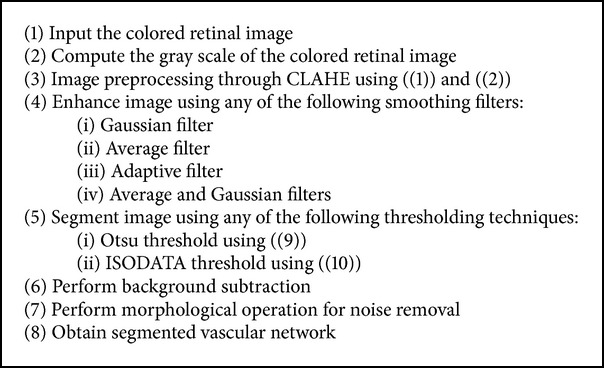
Algorithm for CLAHE global-based thresholding technique.

**Algorithm 2:**
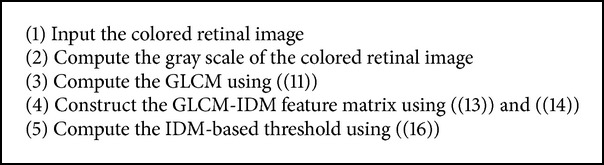
Algorithm for computing IDM-based threshold.

**Algorithm 3:**
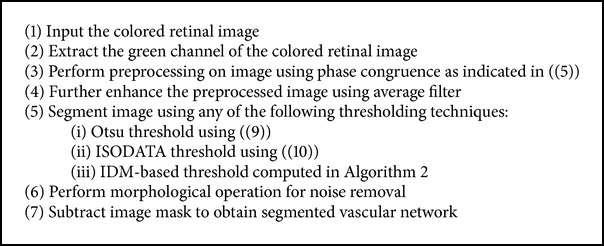
Algorithm for phase congruence global-based thresholding technique.

**Table 1:** Phase congruence parameter description and optimum parameter values.

Parameter description	Parameter symbol	Parameter value (DRIVE)	Parameter value (STARE)
Number of wavelet scales.	*n*_scale_	4	3

Number of filter orientations.	norient	6	5

Wavelength of smallest scale filter.	minWaveLength	3	2.5

Scaling factor between successive filters.	mult	2.1	2.9

Ratio of the standard deviation of the Gaussian describing the log Gabor filter's transfer function in the frequency domain to the filter center frequency.	sigmaOnf	0.55	1.5

Ratio of angular interval between filter orientations and the standard deviation of the angular Gaussian function used to construct filters in the frequency plane.	dThetaOnSigma	1.2	1.7

Number of standard deviations of the noise energy beyond the mean at which we set the noise threshold point.	*k*	2.3	3

The fractional measure of frequency spread below which phase congruency values get penalized.	cutOff	0.5	0.5

Controls the sharpness of the transition in the sigmoid function used to weight phase congruency for frequency spread.	*g*	10	14

**Table 2:** optimal parameter values for CLAHE global thresholding approaches.

Filtering	Filter	CLAHE	CLAHE
Technique	Window size	Clip-limits	Number of tiles
Adaptive filter	4 × 4	0.05	75 × 75
Average filter	3 × 3	0.05	75 × 75
Gaussian filter (sigma = 0.5)	3 × 3	0.04	75 × 75

**Table 3:** Performance of different segmentation methods on DRIVE database.

Preprocessing method	Postprocessing method	Average sensitivity	Average specificity	Average accuracy
CLAHE with average filter using Otsu	Morphological opening	0.57309	0.99048	0.95376
	Morphological opening and median filter	0.59957	0.98439	0.95054
	All techniques combined	0.65349	0.97798	0.94943

CLAHE with Gaussian filter using Otsu	Morphological opening	0.56045	0.99247	0.95449
	Morphological opening and median filter	0.61246	0.98482	0.95204
	All techniques combined	0.67290	0.97651	0.94980

CLAHE with average and Gaussian filters using Otsu	Morphological opening	0.57529	0.98889	0.95255
	Morphological opening and median filter	0.58221	0.98201	0.94688
	All techniques combined	0.64159	0.97172	0.94269

CLAHE with adaptive filter using Otsu	Morphological opening	0.53973	0.99087	0.95118
	Morphological opening and median filter	0.56335	0.98390	0.94691
	All techniques combined	0.61596	0.96773	0.93678

CLAHE with average filter using ISODATA	Morphological opening	0.56730	0.99092	0.95366
	Morphological opening and median filter	0.59380	0.98495	0.95056
	All techniques combined	0.61630	0.98395	0.95162

CLAHE with Gaussian filter using ISODATA	Morphological opening	0.60458	0.98892	0.95508
	Morphological opening and median filter	0.63350	0.98193	0.95125
	All techniques combined	0.67011	0.97695	0.94997

CLAHE with average and Gaussian filters using ISODATA	Morphological opening	0.55402	0.99129	0.95286
	Morphological opening and median filter	0.58024	0.98247	0.94993
	All techniques combined	0.60265	0.98459	0.95104

CLAHE with adaptive filter using ISODATA	Morphological opening	0.58487	0.98978	0.95417
	Morphological opening and median filter	0.61775	0.98318	0.95104
	All techniques combined	0.64348	0.98185	0.95209

Phase congruence with Otsu	Morphological opening	0.45610	0.99210	0.94509
	Morphological opening and median filter	0.48690	0.98754	0.94364
	All techniques combined	0.47232	0.98849	0.94324

Phase congruence with ISODATA	Morphological opening	0.51579	0.98872	0.94722
	Morphological opening and median filter	0.55410	0.98278	0.94517
	All techniques combined	0.53369	0.98459	0.94504

Phase congruence with IDM-based threshold	Morphological opening	0.71520	0.96496	0.94302
	Morphological opening and median filter	0.74247	0.95461	0.93596
	All techniques combined	0.73910	0.95687	0.93772

**Table 4:** Performance of different segmentation methods on DRIVE database.

Method	Average accuracy	Average sensitivity	Average specificity
Human observer	0.9473	0.7761	0.9725
Chaudhuri et al. [6]	0.8773	0.3357	0.9794
Staal et al. [14]	0.9442	0.7345	0.9773
Niemeijer et al. [20]	0.9416	0.7145	0.9801
Zana and Klein [18]	0.9377	0.6971	0.9769
Jiang and Mojon [11]	0.9212	0.6399	0.9625
Marín et al. [19]	0.9452	N/A	N/A
Ricci and Perfetti [24]	0.9646	N/A	N/A
Martinez-Perez et al. [17]	0.9181	0.6389	0.9496
Soares et al. [13]	0.9466	N/A	N/A
Vlachos and Dermatas [26]	0.9285	0.7468	0.9551
Akram and Khan [42]	0.9469	N/A	N/A
Amin and Hong [37]	0.9191	0.6608	N/A
Mendonça and Campilho [28]	0.9463	0.7315	N/A
Tagore et al. [38]	0.9424	N/A	N/A
CLAHE and average filter (ATC) using Otsu	**0.9494 **	**0.6535 **	**0.9780 **
CLAHE and Gaussian filter (ATC) using Otsu	**0.9498 **	**0.6729 **	**0.9765 **
CLAHE with average and Gaussian filters (ATC) using Otsu	**0.9427 **	**0.6416 **	**0.9717 **
CLAHE and adaptive filter (ATC) using Otsu	**0.9368 **	**0.6160 **	**0.9677 **
CLAHE and average filter (ATC) using ISODATA	**0.9516 **	**0.6163 **	**0.9780 **
CLAHE and Gaussian filter (ATC) using ISODATA	**0.9500 **	**0.6701 **	**0.9770 **
CLAHE with average and Gaussian filters (ATC) using ISODATA	**0.9510 **	**0.6027 **	**0.9846 **
CLAHE and adaptive filter (ATC) using ISODATA	**0.9521 **	**0.6435 **	**0.9819 **
Phase congruence with IDM-based threshold (MO)	**0.9430 **	**0.7152 **	**0.9650 **
Phase congruence with IDM-based threshold (MOMF)	**0.9360 **	**0.7425 **	**0.9546 **
Phase congruence with IDM-based threshold (ATC)	**0.9377 **	**0.7391 **	**0.9569 **

**Table 5:** Performance of different proposed segmentation methods on STARE database.

Method	Average accuracy	Average sensitivity	Average specificity
Human observer	0.9354	0.8949	N/A
Hoover et al. [8]	0.9275	0.6751	0.9567
Staal et al. [14]	0.9516	0.6970	N/A
Jiang and Mojon [11]	0.9009	N/A	N/A
Marín et al. [19]	0.9526	N/A	N/A
Ricci and Perfetti [24]	0.9646	N/A	N/A
Soares et al. [13]	0.9480	N/A	N/A
Akram and Khan [42]	0.9502	N/A	N/A
Amin and Hong [37]	0.9081	0.7261	N/A
Mendonça and Campilho [28]	0.9479	0.7123	N/A
Tagore et al. [38]	0.9497	N/A	N/A
CLAHE and average filter (ATC) using Otsu	**0.9409 **	**0.6258 **	**0.9662 **
CLAHE and Gaussian filter (ATC) using Otsu	**0.9435 **	**0.6138 **	**0.9698 **
CLAHE with average and Gaussian filters (ATC) using Otsu	**0.9468 **	**0.6144 **	**0.9735 **
CLAHE and adaptive filter (ATC) using Otsu	**0.9456 **	**0.6135 **	**0.9722 **
CLAHE and average filter (ATC) using ISODATA	**0.9421 **	**0.6238 **	**0.9676 **
CLAHE and Gaussian filter (ATC) using ISODATA	**0.9442 **	**0.6099 **	**0.9709 **
CLAHE with average and Gaussian filters (ATC) using ISODATA	**0.9471 **	**0.6127 **	**0.9740 **
CLAHE and adaptive filter (ATC) using ISODATA	**0.9458 **	**0.6115 **	**0.9726 **
Phase congruence with IDM-based threshold (MO)	**0.9340 **	**0.5202 **	**0.9682 **
Phase congruence with IDM-based threshold (MOMF)	**0.9318 **	**0.5036 **	**0.9671 **
Phase congruence with IDM-based threshold (ATC)	**0.9221 **	**0.5031 **	**0.9567 **

## References

[1] World Health Organization: Prevention of Blindness and Visual Impairment. http://www.who.int/blindness/causes/priority/en/index8.html.

[2] Klonoff D. C., Schwartz D. M. (2000). An economic analysis of interventions for diabetes. *Diabetes Care*.

[3] Chutatape O., Zheng L., Krishman S. M. Retinal blood vessel detection and tracking by matched Gaussian and Kalman filters.

[4] Gagnon L., Lalonde M., Beaulieu M., Boucher M. C. Procedure to detect anatomical structures in optical fundus images.

[5] Tolias Y. A., Panas S. M. (1998). A fuzzy vessel tracking algorithm for retinal images based on fuzzy clustering. *IEEE Transactions on Medical Imaging*.

[6] Chaudhuri S., Chatterjee S., Katz N., Nelson M., Goldbaum M. (1989). Detection of blood vessels in retinal images using two-dimensional matched filters. *IEEE Transactions on Medical Imaging*.

[7] Gang L., Chutatape O., Krishnan S. M. (2002). Detection and measurement of retinal vessels in fundus images using amplitude modified second-order Gaussian filter. *IEEE Transactions on Biomedical Engineering*.

[8] Hoover A., Kouznetsova V., Goldbaum M. (2000). Locating blood vessels in retinal images by piecewise threshold probing of a matched filter response. *IEEE Transactions on Medical Imaging*.

[9] Walter T., Klein J. C., Crespo J., Maojo V., Martin F. (2001). Segmentation of color fundus images of the human retina: detection of the optic disc and the vascular tree using morphological techniques. *Medical Data Analysis*.

[10] Xu L., Luo S. (2010). A novel method for blood vessel detection from retinal images. *BioMedical Engineering Online*.

[11] Jiang X., Mojon D. (2003). Adaptive local thresholding by verification-based multithreshold probing with application to vessel detection in retinal images. *IEEE Transactions on Pattern Analysis and Machine Intelligence*.

[12] Sinthanayothin C., Boyce J. F., Williamson T. H. (2002). Automated detection of diabetic retinopathy on digital fundus images. *Diabetic Medicine*.

[13] Soares J. V. B., Leandro J. J. G., Cesar R. M., Jelinek H. F., Cree M. J. (2006). Retinal vessel segmentation using the 2-D Gabor wavelet and supervised classification. *IEEE Transactions on Medical Imaging*.

[14] Staal J., Abràmoff M. D., Niemeijer M., Viergever M. A., van Ginneken B. (2004). Ridge-based vessel segmentation in color images of the retina. *IEEE Transactions on Medical Imaging*.

[15] You X., Peng Q., Yuan Y., Cheung Y.-M., Lei J. (2011). Segmentation of retinal blood vessels using the radial projection and semi-supervised approach. *Pattern Recognition*.

[16] Zhang B., Zhang L., Karray F. (2010). Retinal vessel extraction by matched filter with first-order derivative of gaussian. *Computers in Biology and Medicine*.

[17] Martinez-Perez M. E., Hughes A., Stanton A., Thom S., Bharath A., Parker K., Taylor C., Colchester A. (1999). Scale-space analysis for the characterisation of retinal blood vessels. *Medical Image Computing and Computer-Assisted Intervention—MICCAI99*.

[18] Zana F., Klein J.-C. (2001). Segmentation of vessel-like patterns using mathematical morphology and curvature evaluation. *IEEE Transactions on Image Processing*.

[19] Marín D., Aquino A., Gegúndez-Arias M. E., Bravo J. M. (2011). A new supervised method for blood vessel segmentation in retinal images by using gray-level and moment invariants-based features. *IEEE Transactions on Medical Imaging*.

[20] Niemeijer M., Staal J., van Ginneken B., Loog M., Abramoff M. D. (2004). Comparative study of retinal vessel segmentation methods on a new publicly available database. *Medical Imaging*.

[21] Fraz M. M., Remagnino P., Hoppe A. (2012). An ensemble classification-based approach applied to retinal blood vessel segmentation. *IEEE Transactions on Biomedical Engineering*.

[22] Fraz M. M., Rudnicka A. R., Owen C. G., Barman S. A. (2014). Delineation of blood vessels in pediatric retinal images using decision trees-based ensemble classification. *International Journal of Computer Assisted Radiology and Surgery*.

[23] Lupaşcu C. A., Tegolo D., Trucco E. (2010). FABC: retinal vessel segmentation using AdaBoost. *IEEE Transactions on Information Technology in Biomedicine*.

[24] Ricci E., Perfetti R. (2007). Retinal blood vessel segmentation using line operators and support vector classification. *IEEE Transactions on Medical Imaging*.

[25] Szpak Z. L., Tapamo J. R. (2008). Automatic and interactive retinal vessel segmentation. *South African Computer Journal*.

[26] Vlachos M., Dermatas E. (2010). Multi-scale retinal vessel segmentation using line tracking. *Computerized Medical Imaging and Graphics*.

[27] Wang Y., Ji G., Lin P., Trucco E. (2013). Retinal vessel segmentation using multiwavelet kernels and multiscale hierarchical decomposition. *Pattern Recognition*.

[28] Mendonça A. M., Campilho A. (2006). Segmentation of retinal blood vessels by combining the detection of centerlines and morphological reconstruction. *IEEE Transactions on Medical Imaging*.

[29] Xiao Z., Adel M., Bourennane S. (2013). Bayesian method with spatial constraint for retinal vessel segmentation. *Computational and Mathematical Methods in Medicine*.

[30] Yin Y., Adel M., Bourennane S. (2013). Automatic segmentation and measurement of vasculature in retinal fundus images using probabilistic formulation. *Computational and Mathematical Methods in Medicine*.

[31] Lupascu C. A., Tegolo D. (2011). Automatic unsupervised segmentation of retinal vessels using self-organizing maps and *k*-means clustering. *Computational Intelligence Methods for Bioinformatics and Biostatistics*.

[32] Lupacu C. A., Tegolo D. (2011). Stable automatic unsupervised segmen tation of retinal vessels using self-organizing maps and a modified fuzzy C-means clustering. *Fuzzy Logic and Applications*.

[33] Saffarzadeh V. M., Osareh A., Shadgar B. (2014). Vessel segmentation in retinal images using multi-scale line operator and K-means clustering. *Journal of Medical Signals and Sensors*.

[34] Setiawan A. W., Mengko T. R., Santoso O. S., Suksmono A. B. Color retinal image enhancement using CLAHE.

[35] Kovesi P. (1999). Image features from phase congruency. *Videre: Journal of Computer Vision Research*.

[36] Kovesi P. Phase congruency detects corners and edges.

[37] Amin M. A., Hong Y. (2011). High speed detection of retinal blood vessels in fundus image using phase congruency. *Soft Computing*.

[38] Tagore M. R. N., Kande G. B., Rao E. V. K., Rao B. P. Segmentation of retinal vasculature using phase congruency and hierarchical clustering.

[39] Zhu T. (2010). Fourier cross-sectional profile for vessel detection on retinal images. *Computerized Medical Imaging and Graphics*.

[40] Otsu N. (1979). A threshold selection method from gray level histograms. *IEEE Transactions on Systems, Man and Cybernetics*.

[41] Research Section (2013). *Digital Retinal Image for Vessel Extraction (DRIVE) Database*.

[42] Akram M. U., Khan S. A. (2013). Multilayered thresholding-based blood vessel segmentation for screening of diabetic retinopathy. *Engineering with Computers*.

